# Belonging, happiness, freedom and empowerment—a qualitative study of patients’ understanding of health in early rheumatoid arthritis

**DOI:** 10.1186/s41927-024-00399-2

**Published:** 2024-06-27

**Authors:** Ellen Landgren, Elisabeth Mogard, Ann Bremander, Elisabet Lindqvist, Maria Nylander, Ingrid Larsson

**Affiliations:** 1 Department of Clinical Sciences, Section of Rheumatology, Lund University, Lund, Sweden; 2 Department of Rheumatology, Skåne University Hospital, Lund, SE-221 85 Sweden; 3 Spenshult Research and Development Centre, Halmstad, Sweden; 4 Department of Regional Health Research, University of Southern Denmark, Odense, Denmark; 5 Danish Hospital for Rheumatic Diseases, University Hospital of Southern Denmark, Sonderborg, Denmark; 6 Swedish Rheumatism Association, Stockholm, Sweden; 7 School of Health and Welfare, Halmstad University, Halmstad, Sweden

**Keywords:** Health, Interviews, Patients, Phenomenography, Rheumatoid arthritis

## Abstract

**Background:**

Rheumatoid arthritis (RA) is a chronic, systemic, inflammatory joint disease, that influences patients’ health in different ways, including physical, social, emotional, and psychological aspects. The goal of rheumatology care is to achieve optimal health and personalised care and therefore, it is essential to understand what health means for patients in the early course of RA. The aim of this study was to describe the understanding of health among patients with early RA.

**Methods:**

The study had a descriptive qualitative design with a phenomenographic approach. Phenomenography is used to analyse, describe, and understand various ways people understand or experience a phenomenon, in this study, patients’ understandings of health. Individual semi-structured interviews were conducted with 31 patients (22 women and nine men, aged (38–80) with early RA, defined as a disease duration of < 1 year, and disease-modifying anti-rheumatic drugs (DMARDs) for 3–7 months. The phenomenographic analysis was conducted in 7 steps, and the outcome space presents the variation in understanding and the interrelation among categories. In accordance with the European Alliance of Associations for Rheumatology’s (EULAR) recommendations, a patient research partner participated in all phases of the study.

**Results:**

The analysis revealed four main descriptive categories: ‘Health as belonging’ was described as experiencing a sense of coherence. ‘Health as happiness’ was understood as feeling joy in everyday life. ‘Health as freedom’ was understood as feeling independent. ‘Health as empowerment’ was understood as feeling capable. Essential health aspects in early RA are comprised of a sense of coherence, joy, independence, and the capability to manage everyday life.

**Conclusions:**

This study revealed that patients’ perception of health in early RA encompasses various facets, including a sense of belonging, happiness, freedom, and empowerment. It highlighted that health is multifaceted and personal, emphasizing the importance of acknowledging this diversity in providing person-centred care. The findings can guide healthcare professionals to deepen patients’ participation in treatment goals, which may lead to better treatment adherence and health outcomes.

**Supplementary Information:**

The online version contains supplementary material available at 10.1186/s41927-024-00399-2.

## Background

Rheumatoid arthritis (RA) is a chronic, systemic, inflammatory joint disease with a prevalence of 0.5–1%. RA is characterised by progressive symmetric inflammation of affected joints, resulting in cartilage destruction and disability. The disease affects more women than men and occurs in all ages but is more frequent around middle age [1]. Early RA symptoms are characterised by morning stiffness, swollen and tender joints, fatigue, and generalised sickness affecting the patients’ overall health. Early diagnosis and treatment are important for a better prognosis. The treatment target is to reach remission or at least significantly lower disease activity [2]. The importance of prompt and targeted treatment is underlined by the fact that the disease may affect patients’ ability to work, their economic situation, and their overall quality of life [3]. In addition to the pharmacological Disease Modifying Anti-Rheumatic Drug treatment (DMARD), patients should have access to non-pharmacological treatment—including nursing consultations, physical therapy, occupational therapy, patient counselling regarding lifestyle habits, disease- and treatment information, coping strategies, and promotion of self-management—as adjunct interventions to learn how to manage the disease [4, 5]. According to the European Alliance of Associations for Rheumatology (EULAR), the treatment goal for patients with RA should aim for the best care and optimal health, and should be based on shared decision-making between patients and healthcare professionals [6] and include person-centred care [5]. Person-centred care is respectful, empowering, and adopts the person’s perspective. This approach empowers patients to take greater responsibility for their treatment. Empowered and educated patients manage their disease better and co-operate with healthcare professionals to maintain or restore their health status [7].

Even though effective treatments are accessible, the disease can affect patients’ health in different ways, including physical, social, emotional, and psychological aspects [8]. Symptoms can lead to an inability to participate in desired activities and fulfil social roles [9]. Being able to perform activities is positively associated with mental and physical health in patients with RA [10], and those experiencing participation restrictions tend to report increased fatigue, disability, and pain [11]. In contrast, patients who can participate in social activities are more comfortable managing their disease [10]. Facilitating participation in daily activities is an important aspect of rehabilitation, with participation often linked to a sense of belonging and engagement in activities like work or leisure [12]. Social relationships play an essential role in mental health [13] and it is essential for patients with RA to maintain these relationships and continue participating in activities [14]. To achieve holistic person-centred care, EULAR recommends a biopsychosocial model that includes biological, social, psychological, and behavioural dimensions of illness in patient assessment, treatment, and care [15, 16]. This is consistent with the World Health Organisation’s (WHO) definition, which states that health is a positive concept emphasising social, personal, and physical assets and should be seen as a resource in everyday life (a process) and not the object of living (a state) [17]. According to the WHO constitution, reaching the highest possible level of health is one of the fundamental rights of every human being [18]. Other descriptions of health include health-related abilities and well-being, two dimensions with a causal effect on health [19]. Having the capability to adapt and self-manage, is another, dynamic description of health, which is based on the individual’s capacity to cope, and maintain or restore one’s integrity and sense of well-being [20]. Informed opinions and active cooperation between patients and healthcare professionals are important aspects in improving health [18, 21].

A systematic review shows that patients with RA request support to achieve normalcy, maintain wellness, and maintain the same roles and expectations as prior disease [22]. Work is often a prioritised part of everyday life and contributes to well-being and a sense of normality in living with RA [23]. Patients with established RA describe health as being able to function normally, experience well-being, have a healthy lifestyle, and be free from disease [24]. In early RA, however, qualitative studies regarding patients’ understanding of health are lacking. Despite prompt initiation of effective therapy and tight control, patients still experience unmet needs, such as a lack of communication and patient empowerment, and symptoms affecting the psychosocial, physical, and mental aspects of health [25–27]. There are international recommendations regarding treatment goals for RA focusing on disease activity and function [21]. How these goals align with patients’ understanding of health early in the disease course of RA is lacking. With increased knowledge of patients’ understanding of health relevant advice and more targeted person-centred care can be provided for newly diagnosed patients. Therefore, the aim of this study was to describe the understanding of health among patients with early RA.

## Methods

### Design

This study had a descriptive, qualitative design and is based on a phenomenographic approach [28]. Phenomenography is an approach used to analyse, describe, and understand different ways of experiencing a phenomenon in the surrounding world. A distinction is made between the first-order perspective—the external perspective, how a phenomenon really is—and the second-order perspective, the subjective perspective, how a phenomenon is perceived by others. Phenomenography describes experiences from the second-order perspective [29]; in this study, patients’ understandings of health. This study was conducted in collaboration with a patient research partner (MN), aiming to improve the relevance, quality, and validity of the research process, which aligns with EULAR recommendations for clinical research [30, 31]. The patient research partner was involved in the study from the beginning to include patients’ perspectives throughout the research process. The patient research partner has participated in the work with the study design, giving feedback on the interview questions, participated in the analysis processes, and given critical input on the manuscript, and therefore is a co-author of the manuscript. To ensure trustworthiness, the study fulfilled the consolidated criteria for reporting qualitative research’s (COREQ) 32-item checklist [32].

### Participants

A purposeful sampling was used to include patients of different sexes, ages, and different living areas in order to find variations and describe different ways of understanding health [33]. The inclusion criteria were: a diagnosis of RA according to the American College of Rheumatology/European League Against Rheumatism’s 2010 criteria [34]; disease duration of ≤ 1 year; DMARD treatment for 3–7 months; ≥18 years of age, and being able to speak, read, and write in Swedish. Patients were invited to participate in the present study by their rheumatologist or a nurse at their rheumatology clinic. A total of 33 patients were invited and two declined due to personal reasons. Additional information about the study, both in oral and written form, was given by the first or the last author before the interview (EL or IL). A total of 31 patients; 22 women, and 9 men, aged 38–80 years, from two rheumatology clinics in four cities, representing both university hospitals and regional rheumatology specialist outpatient clinics in southern Sweden, participated in this study. All patients were treated according to standard clinical practice with early DMARD treatment, which followed the National Pharmacological Guidelines for RA [35]. The patients’ characteristics are presented in Table 1.

**Table 1:** Participant demographic, clinical, and self-reported characteristics

Number of participants (*n*)	31
Site of recruitment Regional Rheumatology Specialist Outpatient Clinic/University Hospital (n)	3/28
Individual interviews (n)	31
Gender Female/Male (n)	22/9
Age (years) Median (range)	56 (38–80)
Disease Duration (months) Median (range)	5 (3–9)
DMARD Treatment Duration (months) Median (range)	5 (3–7)
Current DMARD treatment (n) csDMARDs bDMARDs Discontinued treatment	30 8 1
Civil Status (n) Co-habiting/Living alone	27/4
Education Level (n) Primary School/Secondary/University	8/15/8
Employment (n) Employed/Student/Unemployed/Retired	14/1/3/13
NRS Pain (mm)* Median (Range)	27 (0–70)
NRS General Health (mm)* Median (Range)	26 (0–80)
NRS Fatigue (mm)* Median (Range)	30 (0–95)

*during the past week. Range 0–100 (best to worst)

*Abbreviations*: csDMARD(s): conventional synthetic disease-modifying anti-rheumatic drug, bDMARD: biological disease-modifying anti-rheumatic drug, NRS: numeric rating scale

### Data collection

Individual interviews were conducted with 31 patients from 2017 to 2018 by the first and last authors (EL, IL), both nurses with competence in rheumatology and without treatment positions and no previous relation to the patients. Data collection was performed in a close collaboration between the interviewers and the last author is an experienced senior qualitative researcher. The first author was a PhD student at the time of the study, trained in qualitative interviewing. Two pilot interviews were performed to test the quality of the interviews, which were included in the study because no adjustments were required. Inclusion of patients continued until data saturation was reached meaning that data collection continued until no new information was obtained, and enough in-depth data was available to illuminate patterns of the phenomenon [36]. A semi-structured interview guide was developed for this study (supplementary file 1), and included different topics, the first topic of which was health. Questions used to elicit the understanding of health included “What is health for you?”, “What does health mean to you?”, “Do you experience that your health has changed since you were diagnosed with RA?” The responses were followed by probing questions such as “Can you tell me more about that?” “Can you elaborate on that?”, “What do you mean by that?”, “Can you describe it in more words?” The interviews lasted between 16 and 127 min, with a median of 43 min and a total interview length of 26 h and 9 min. The interviews were conducted in an undisturbed room at the rheumatology clinics or at a research and development centre, with solely the interviewer and the participant present during the interview. Two participants chose to be interviewed at home. The interviews were digitally recorded and transcribed verbatim.

### Data analysis

**Fig. 1:**
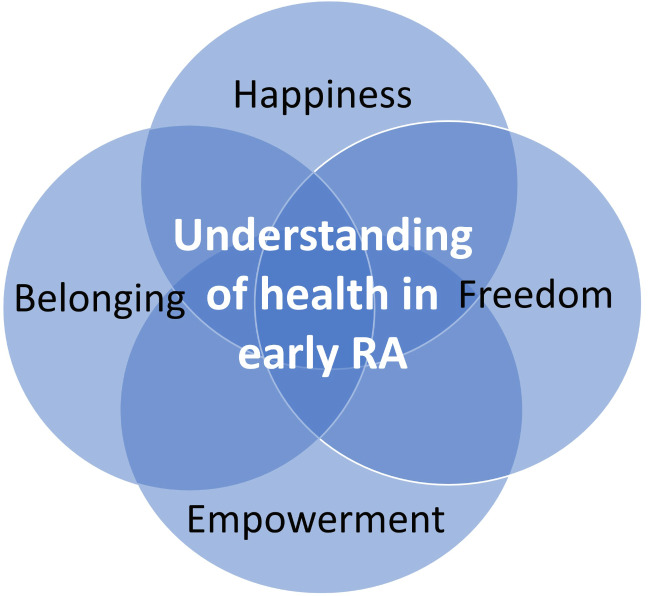
The outcome space—understanding of health among patients with early RA and the relationship between categories

The analysis was conducted in accordance with Larsson and Holmström’s (2007) seven steps of phenomenographic analysis: (1) The transcripts were read thoroughly. (2) The transcripts were re-read and marked where the interviewee gave answers pertaining to the main research questions; in this case ‘What does health mean to you?’ and marking quotes. (3) Identifying how patients described health and a preliminary description of each category was made (4) Grouping the descriptions of health into categories based on similarities and differences. (5) Comparing categories, looking for non-dominant ways of understanding health. (6) Looking for relations between the categories and finding a structure in the outcome space (Fig. 1). The outcome space constitutes the relations between the categories. (7) Assigning a metaphor to each category of health [37]. A qualitative software programme, NVivo 1.7.1 (QSR International, London, UK) was used as an administrative tool, but the automatic analysis functions were not used. An example illustrating the analysis is provided in Table 2.

**Table 2:** An example illustrating the analysis

Quote	Dominant elements	Preliminary description	Categories	Metaphor
I am the kind of person who likes to work. Maybe there are people who can’t…bear to work or something like that. But I’ve always been someone who, I wanted to have a meaningful life. I wanted to do something for others. I loved my profession	To have a meaningful life	Belonging	Feeling a sense of coherence	Health as belonging
I love going to restaurants and going out to eat good food. It’s health, doing things you enjoy that make you feel good mentally in addition to the physical, I think that’s great. To be able to be spontaneous in one’s activities, that is health, to be able to spend time with friends, to eat healthy, to not be in so much pain that it limits me too much in what I want to do	Engaging in activities that bring joy and promote both mental and physical well-being	Enjoying life	Feeling joy in everyday life	Health as happiness
Health for me is that I can actually move, I bicycle a lot and I walk a lot, health to me is to be able to do that, to be independent	Being autonomous and independent of other people	Managing everyday life independently	Feeling independence	Health as freedom
I still go to Zumba, I run, I bicycle…I am more aware that I have to exercise. I have to do something. I have to do it for my own sake. To think like that, I have to help my body, it’s like my body is having a battle, I have to help it somehow, I can’t just sit and be lazy, I have to do something	Having the inner strength to exercise and take control of life	Taking control of life	Feeling capable	Health as empowerment

The analysis was first conducted by the first author EL, and the last author IL acted as a co-assessor. The analysis was then discussed with the co-authors (AB, EM, EL) and the patient research partner (MN). The analysis was conducted in Swedish and was translated to English afterward. A language review was conducted by a professional interpreter, and all the authors reviewed and accepted the final manuscript.

## Results

The results showed four ways of understanding health among patients with early RA, with a metaphor assigned to each one; ‘health as belonging’, ‘health as happiness’, ‘health as freedom’, and ‘health as empowerment’. The results describe the significant features of each category exemplified with quotations from the interviews. The second part of the results section contrasts the internal relations between the categories, described as the outcome space.

### Health as belonging

A characteristic feature of the category—‘health as belonging’—was to experience a sense of coherence: a meaningful day and being needed. This was expressed as maintaining work capacity and participating in family life and leisure. Working gave structure and purpose to the day. Combining the ability to fulfil one’s work ethic and not focus on the disease generated positive health outcomes.

*“I am the kind of person who likes to work. Maybe there are people who can’t…bear to work or something like that. But I’ve always been someone who, I wanted to have a meaningful life. I wanted to do something for others. I loved my profession.”* Woman, 56 years old

Experiencing flare-ups and increased symptoms limited patients’ ability to commit to activities, which led to a restricted life. Patients described how animals contributed to the experience of belonging and a meaningful life. An animal represented responsibility and gave structure to the day as well as motivation for physical activity. It was also a source of love, joy, and belonging. To be able to maintain a sense of coherence and to participate in family life and desired activities was perceived as healthy and led to the confirmation of being needed.

*“We have grandchildren now. And that makes you feel good, even if it’s a bit hard too (laughing). But it certainly makes you feel good! It is a great joy! And you feel that you can be useful too”* Woman, 72 years old.

### Health as happiness

‘Health as happiness’ was understood as feeling joy in everyday life. Features of this category were enjoyment and a lust for life. Health was understood as a foundation for feelings of joy and excitement, and a lack of health led to unease. Happiness was having fun, cooking food, enjoying dinner with friends, and participating in hobbies and children’s activities. Flare-ups and symptoms negatively affected well-being and suppressed patients’ mood and lust for life.

“*I love going to restaurants and going out to eat good food. It’s health, doing things you enjoy that make you feel good mentally in addition to the physical, I think that’s great. To be able to be spontaneous in one’s activities, that is health, to be able to spend time with friends, to eat healthy, to not be in so much pain that it limits me too much in what I want to do” Woman, 63 years old*

### Health as freedom

‘Health as freedom’ was described as feeling independent, which meant living without physical, financial, and mental restraints. Patients described a spectrum between ability and incapability, where health was understood as independence. To be able to participate in desired activities and live with autonomy and independence despite the disease were essential aspects of health. Increased symptoms restricted the possibility to fulfil life goals, but with low disease activity, it was possible to experience health and independence in everyday life.

“*Health for me is that I can actually move, I bicycle a lot and I walk a lot, health to me is to be able to do that, to be independent*” Woman, 63 years old

Patients described a stable economic situation and being able to work as important aspects of experiencing health as freedom, while sick leave led to worry and a confined life. A stable disease enabled workability and a stable economic situation.

*“Actually, health is money too. Because if you have no finances, you worry and then your health is not good” Man, 51 years old*

Patients described the mental aspect of health as freedom, as having the ability to think positively and to keep up the faith about the future. Worrying about disease prognosis and the future affected patients’ well-being and the possibility to experience independence and freedom.

### Health as empowerment

‘Health as empowerment’ was understood as feeling capable and having the knowledge and ability to self-manage one’s health and life situation. Maintaining a positive attitude and using the power of thought were described as positively affecting one’s experience of health as empowerment. The perceived possibilities to affect ones’ health included lifestyle changes: smoking cessation, reduced alcohol intake, and striving for a healthy weight through a healthy diet and a physically active life. Patients described striving for a healthier lifestyle as a struggle, but education encouraged healthy lifestyle choices, and supported empowerment. Health as empowerment was described as being in charge of one’s life and disease management. Confidence and knowledge did not explicitly lead to a healthier lifestyle, and patients appreciated support from healthcare professionals.

*“I still go to Zumba, I run, I bicycle…I am more aware that I have to exercise. I have to do something. I have to do it for my own sake. To think like that, I have to help my body, it’s like my body is having a battle, I have to help it somehow, I can’t just sit and be lazy, I have to do something!” Woman, 44 years old*

### Outcome space

The four categories of understanding health among patients with early RA and their relations constituted the outcome space and represented the variation of the patients’ collective understanding of health (Fig. 1). The categories have no hierarchical relation but are linked to one another in different ways and can be regarded as a structure for describing variation.

‘Health as belonging’ and ‘health as happiness’ were related, since coherence led to feelings of satisfaction and joy. Another unifying factor in the meaning of health was participation, which both led to happiness and belonging and was also linked to ‘health as freedom’. Experiences of freedom led to participation in activities that sparked joy and brought meaning to life. Reduced income due to sick leave could lead to worry, sadness, and cancelled plans, connecting ‘health as freedom’, ‘belonging’, and ‘happiness’.

‘Health as empowerment’ was related to ‘health as happiness’, since information encouraged capability and health education could ease worry and anxiety. Experiences of mental and physical power and being able to live independently connected ‘Health as empowerment’ and ‘health as freedom’. ‘Health as empowerment’ was also connected to ‘health as belonging’ through the positive exchange of healthy lifestyle changes together with others and managing life and disease.

## Discussion

This study describes four different ways of understanding health among patients with early RA; ‘health as belonging’, ‘happiness’, ‘freedom’, and ‘empowerment’. Essential aspects of health were a sense of coherence, joy, independence, and the capability to manage everyday life. This is related to WHO’s (1986) description of health as a positive concept that highlights the significance of social and personal assets alongside physical capacities. Patients with early RA perceived health as including social resources described as ‘belonging’ and personal resources in the shape of happiness. Health was also described as having physical capacities, expressed as being free and independent, and inner capacity, in terms of empowerment. The result shows how health in early RA consists of physical, psychological, social, and, to some extent, existential aspects. Previous research examining the understanding of health and quality of life among patients with established RA was primarily associated with functioning normally, experiencing well-being, and having a healthy lifestyle [24].

‘Health as belonging’ was understood as feeling a sense of coherence that included the benefits of participating in activities and feeling needed. Patients reported positive experiences of belonging and participation in a social context [38], and negative influences on relationships when the disease limits the ability to care for others [9]. Social resources are important aspects of health and quality of life [17] and are essential parts of the biopsychosocial model and holistic care [16]. In the present study, work was described as an essential part of health. Previous research shows that employment increases self-esteem and a sense of purpose [39], which should be considered in holistic person-centred care [7].

The present study shows that ‘health as happiness’ was understood as feeling joy in everyday life and having a positive spirit. A previous systematic review showed that happiness was conceptualised as a positive health indicator and that general health was associated with general happiness, while suffering from a severe disability was associated with less happiness [40]. Happiness has been associated with longevity and stronger social relations, and years lived by a happy person are more enjoyable and experienced with better health [41, 42].

‘Health as freedom’ was described as experiencing physical, mental, and financial independence, which underlines previous research showing that affected work productivity due to RA leads to reduced quality of life [43] and economic consequences for both the patients and society [23, 44]. Psychological distress and impaired physical functioning are well-known complications of RA that affect health [45]. Physical deterioration and functional losses also affect patients’ health [46]. Independence was crucial for patients’ health, supported by findings from a previous meta-synthesis indicating that independence was essential in conducting a normal life [8].

In the present study, patients with early RA described health as feeling empowered and having the capability to affect one’s situation and be in charge of one’s life. This corroborates with health theories defining health as the capability to adapt and self-manage [20], suggesting that a strengthened capability to adapt and to self-manage often improves subjective well-being [47]. In health promotion and behaviour change, interventions are more likely to succeed if the person involved chooses targets that concern them. Increased empowerment constitutes an increased quality of life and a higher likelihood of remaining healthy [48]. The results from the present study support the positive effects of health education, previously found in a scoping review that showed increased autonomy and improvement in self-management among patients with RA after receiving person-centred support [49]. The patients in the present study valued health education that supported empowerment. Being confident in self-management and having an understanding relationship with the healthcare provider improves adherence to treatment and shared decision-making [50]. To empower patients and improve their health; person-centred care and shared decision-making should be implemented in clinical practice [51].

### Strengths and limitations

Trustworthiness in qualitative research is defined according to credibility, transferability, confirmability, and dependability [52]. We performed a purposeful sampling of participants to represent both university hospitals and regional settings, and included variation regarding age, sex, and living areas to strengthen transferability. To assure quality and confirmability, the author’s pre-understanding was critically reflected upon to avoid affecting the process with experiences or preconceptions. Dependability was assured through the described steps of analysis and the quotes used to verify the findings. In addition, the interview questions, the analysis steps, and the interpretation process are described and transparent to enhance transferability. A patient research partner participated in all steps of the study to enhance trustworthiness. The fact that most patients were included from university hospitals could be a potential limitation in transferability. The duration of the interviews could also be considered a potential limitation, as some were relatively short. However, 31 interviews were included to reach data saturation, and the interview texts were deemed rich and showed variation and were all included in the analysis.

## Conclusions

This study gives insight into and increases the understanding of health in early RA from the patients’ perspective. Health was understood as ‘belonging’, ‘happiness’, ‘freedom’, and ‘empowerment’. It highlighted that health is multifaceted and personal, emphasizing the importance of acknowledging this diversity in providing person-centred care. The findings can guide healthcare professionals to deepen patients’ participation in treatment goals, which may lead to better treatment adherence and health outcomes.

### Electronic supplementary material

Below is the link to the electronic supplementary material.

Supplementary Material 1

Supplementary Material 2

## Data Availability

The data supporting the results reported in the manuscript are not publicly available as ethical approval for the study requires that the transcribed interviews are kept in locked files, accessible only to the researchers.
