# Supplementary material for: Belonging, happiness, freedom and empowerment—a qualitative study of patients’ understanding of health in early rheumatoid arthritis
Source: BMC Rheumatol. 2024 Jun 27;8:29. doi: 10.1186/s41927-024-00399-2 (PMC11212251; doi:10.1186/s41927-024-00399-2)
Supplement: Supplementary file 1 — Supplementary Material 1 [file 41927_2024_399_MOESM1_ESM.docx]

## Interview guide

What does health mean to you?

How do you perceive your health?

How are you today?

How do you perceive your health have changed since your diagnosis of Rheumatoid Arthritis (RA)?

-How do you handle it?

What do you do to affect your health?

What makes you feel good? What can you for yourself to feel good?

Has life changed since you were diagnosed with Rheumatoid Arthritis (RA)? If it has changed – how?

Exploratory/in-depth questions: Can you tell me more about it? Can you elaborate on that? Can you give an example? Is there anything else you would like to add? How? Can you describe it in more words? What do you mean about...?

At the end of the interview, ”to summarize, you talked about (…) is there anything else you would like to add?”
